# Generalized pustular psoriasis: a multicentric study on patient characteristics and clinical burden

**DOI:** 10.1186/s13023-025-03559-w

**Published:** 2025-02-08

**Authors:** Cristina Bulai Livideanu, Jérémy Gottlieb, Denis Jullien, Thierry Passeron, Sophie Vildy, Emmanuel Delaporte, Carle Paul, Julien Chollet, Marie Najean, Denis San, Charles Taieb, Bénédicte Charles, Emmanuel Mahe, Pierre-André Becherel, Laurent Misery

**Affiliations:** 1 Department of Dermatology, Hôpital Larrey, Toulouse University Hospital, Toulouse, France; 2 Department of Dermatology, Niort, CH France; 3 Department of Dermatology, Faculty of Medicine Lyon-Est, Hôpital Edouard Herriot, University of Lyon, Lyon, France; 4 Department of Dermatology, Université Côte D’Azur, Centre Hospitalier Universitaire Nice, Nice, France; 5 Department of Dermatology, Hôpital privé du Confluent, Nantes, 44200 France; 6 Department of Dermatology, Aix Marseille University, APHM, North Hospital, Marseille, France; 7 Dermatology Department, Toulouse University Hospital and Inserm UMR 1291- CNRS, Infinity (L’Institut Toulousain des Maladies Infectieuses et Inflammatoires), Toulouse, France; 8 Boehringer Ingelheim France, Paris, France; 9 Patients priority, EMMA, Paris, France; 10 France psoriasis, Paris, France; 11 Department of Dermatology, Hôpital Victor Dupouy, 69 rue du Lieutenant-Colonel Prud’hon, Argenteuil, 95100 France; 12 Dermatology Department, Private Hospital Antony, Paris, France; 13 Univ Brest, LIEN, Brest, France; 14 Department of Dermatology, Venereology and Allergology and French Expert Centre on Pruritus, University Hospital of Brest, Brest, France

**Keywords:** Generalized Pustular Psoriasis, Burden, Quality of life, Stigmatization

## Abstract

The objective of this study was to assess the demographic characteristics and impact on quality of life (QoL) of patients with PPG in France through a multicentre study. The results of the study are as follows: The PRO [PUSH-D, PHQ-9 et GAD-7] revealed that more than half of the patients exhibited a significant impact on their quality of life. High scores for fatigue, stress, skin and joint pain were reported, with 65% of patients at risk of mild to severe depression. The clinical burden was also assessed. A total of 48.8% of patients were hospitalised, while 39% took sick leave. This study is the first to assess the PUSH-D, PHQ-9 and GAD-7 scores in patients with PPG, which highlighted a significant clinical burden and negative impact on their daily lives.

**Dear Editor**

Generalized pustular psoriasis (GPP) is a severe, life-threatening, chronic dermatosis characterized by recurrent episodes of erythematous eruptions with sterile pustules, high fever, fatigue, and neutrophil leukocytosis [1]. It is a rare form of psoriasis with an estimated cumulative prevalence of 45.2 per million in the French population in 2017 [2]. Quality of life (QoL) has been extensively studied in patients with plaque psoriasis but has received minimal attention in patients with GPP. In particular, data from Europe are lacking. Our aim was to assess demographics and impact on QoL of patients with GPP in France in a multicentre study.

A questionnaire was sent to patients followed for their GPP in 10 different dermatology departments in France. Questions focused on patients’ demographics and impact on their QoL. The latter was assessed via different instruments: Dermatology Life Quality Index (DLQI; score from 0 to 30), Patient Unique Stigmatization Holistic tool in Dermatology (PUSH-D; score from 0 to 68), EuroQol-5 Dimensions (EQ-5D) utilities (score from 0 to 100, with 100 representing the best QoL), Patient Health Questionnaire (PHQ-9 score from 0 to 27), and Generalized Anxiety Disorder − 7 (GAD-7 score from 0 to 14), and Visual Analog Scale (VAS score from 0 to 10) for joint pain, skin pain, fatigue, and perceived stress.

Forty-one patients were recruited, mostly women (*n* = 25). The mean (SD) age was 52.3 (15.8) y.o for women and 49.2 (10.4) for men, 34% of patients were obese. The diagnosis of GPP was made at the hospital (by an emergency physician or dermatologist) in 87% of cases, in outpatient setting for the remaining 13%.

In the last 5 years, 21.7% of participants stated to have consulted a psychologist for their GPP with an average (SD) of 8.2 (5.4) annual visits, 39% stated to have taken a sick leave for their GPP with an average (SD) of 26 (5.2) days per leave and 48.8% stated to have been hospitalized for their GPP with an average (SD) of 5.2 (4.7) days per hospitalization. 83.3% stated moderate to severe fatigue, including 63.9% of all patients even reporting severe fatigue. 82.9% stated moderate to severe stress (including 42.9% of all patients reporting severe stress). 65.5% stated moderate to severe skin pain (including 34.5% of all patients reporting severe skin pain). Finally, 63.3% reported moderate to severe joint pain and (including 26.7% of all patients reporting severe joint pain). Their QoL was also assessed using Patient-Reported Outcome Measures (PROM): 59% reported an important effect of their GPP on the DLQI, 65% were at risk of mild to severe depression, while 44% reported signs of generalized anxiety disorder (Table 1).

Our study was the first to evaluate the PUSH-D, PHQ-9 and GAD-7 scores in patients with GPP. Like in other studies, GPP was more common in women [3]. While 17% of the population in France is obese [4], the proportion of obese patients in our study was twice as high. Patients with GPP experienced a significant clinical burden that negatively impacted their daily lives. The DLQI score showed that more than half of patients faced an important or very important impact on their QoL, reflecting a greater impact than plaque psoriasis [5, 6]. Moreover, more than a third of patients stated to have taken sick leaves with almost a month per leave [7]. GPP and plaque psoriasis require distinct approaches to their management and treatment. With the advent of new treatments for GPP, it would be interesting to evaluate the effect that new biologics may have on these elements of clinical burden.

**Table 1:** Age and gender of participants & impact of generalized pustular psoriasis on patients

Age and gender of participants							
Age	N	Min	Q1	Mean ± sd	Median	Q3	Max
*At GPP diagnosis*							
Male	13	16	72.5	38.2 ± 15.6	32.5	77.5	67
Female	25	26	32.75	45.8 ± 14.8	43.5	59.25	69
Total *	41	16	31.25	42.4 ± 14.9	40.5	53.5	69
*At the time of the questionnaire*							
Male	13	36	39	49.2 ± 10.4	48	53	70
Female	25	26	42	52.3 ± 15.8	59	66	75
Total *	41	26	42	51.8 ± 14.4	53	62	80
Impact of generalized pustular psoriasis on patients							
Score				Interpretation		N	%
**DLQI** :37patients answered							
< 6				No effect		12	32.43%
between 6 et 10				Moderate effect		3	8.11%
between 11 et 20				Important effect		13	35.14%
≥ 20				Very important effect		9	24.32%
**PUSH-D** : 40 patients answered							
				Mean score : 17 ;7 ± 18.7			
**PHQ-9** : 40 patients answered							
“1–4”				Absence of depression		14	35.00%
“5–9”				Mild depression		15	37.50%
“10–14”				Moderate depression		3	7.50%
> 15				Severe depression		8	20.00%
**Equation** **5-D**:							
EQ-5D utility				Mean score : 0.54 ± 0.35		28	
EQ-5D VAS				Mean score : 55.7 ± 23.2		40	
**GAD 7** : 41 patients answered							
> 7				Presence of generalized anxiety disorder		18	43.9%

## Data Availability

The datasets generated by the survey and/or analysed as part of this study are available at the following address: https://l.ead.me/bfXwAg. The data are compiled in such a way as to respect the anonymity of respondents as required by the Ethics Committee.
